# Experience Is Fallacious and Observation Is Difficult

**Published:** 1872-12

**Authors:** 


Experience is Fallacious and Observation is Difficult. Dr. Montard Martin, of the Hospital Beaujon, in the result of a series of inquiries, instituted by him upon the adaptability of bromide of potassium to the complaints of early childhood, presented to the Académic Impériale de Médicine, in Paris, and published in the *Bulletin Géneral Thérapeutique, November*, 1869, is positive in declaring that the agent "*should not be given where there is diarrhœa.*"

Is it possible that bromide of potassium, administered to children in France, occasions different effects than when administered to children in America; or, does bromide of potassium administered to children of French parentage occasion effects differing from those occasioned by the agent when administered to children of other nationalities? During the same year that I became acquainted with Montard Martin's charge, I also read in several American journals reports of cases of diarrhœa in children, that were intractable until the use of bromide of potassium put a stop to the discharges, and permitted the patients to regain health. The testimony was from more than a single source, as regards locality and reporter, and each furnished representative cases, and concurred in observations establishing *the almost uniform beneficial energy of bromide of potassium in the diarrhœa in infants*, the diarrhœa of suckling babes during dentition, and the diarrhœa of babes artificially nourished, and the diarrhœa of children of any age other than that of two to ten.

For more than two years I have employed the agent somewhat extensively, and almost to the exclusion of any other medicinal agent proper, only causing attention to be paid to the manner of preparing food, and the mode of administering nourishment, and I cannot now recall a single case of failure to perceive the object accomplished, for which the bromide of potassium was administered, whilst I remember several cases, for whose lives the mothers had despaired, where the hopes were revived from the first dose of bromide of potassium, and were fructified in the restoration of their children to perfect health by the sufficient continuance of the agent. Truly, "experience is fallacious," and "observation is difficult."

F. A. R.

Memphis, Tennessee.

